# Case Report: Exploring beyond tumors: intra-abdominal mass in a young patient with Crohn's disease

**DOI:** 10.3389/fped.2025.1544459

**Published:** 2025-05-22

**Authors:** Akash Daswaney, Pratikkumar Patel, Ramanathapura Haricharan, Rehab Mohamed, Kanya Ahuja

**Affiliations:** ^1^ Department of Pediatrics, Charleston Area Medical Center, Charleston, WV, United States; ^2^ Department of Pediatrics, West Virginia University - Charleston Division, Charleston, WV, United States; ^3^ Department of Pathology, West Virginia University - Charleston Division, Charleston, WV, United States

**Keywords:** intraabdominal mass, Crohn's disease, carcinoid tumor, inflammatory bowel disease, abdominal mass

## Abstract

An abdominal mass is an uncommon manifestation of Crohn's disease, often mimicking the granulomatous, infectious, or neoplastic conditions. In this case, a 12-year-old girl presented with abdominal pain and a mass in the right lower quadrant (RLQ). Despite the absence of typical gastrointestinal symptoms, investigations revealed an indeterminate abdominal mass. Colonoscopy revealed a polypoid mass in the cecum, which eventually led to an ileocecectomy. Biopsy results showed noncaseating granulomas, characteristic of Crohn's disease (CD). Laboratory tests indicated anemia, elevated inflammation markers, and positive genetic markers for CD. This case underscores the diagnostic challenge of CD in children, particularly when it presents atypically as a benign mass. It emphasizes the importance of considering inflammatory bowel disease (IBD) in the differential diagnosis of intraluminal intra-abdominal masses and stresses the need for early recognition and treatment to improve outcomes, highlighting the significance of a comprehensive evaluation incorporating symptoms, radiology, and histological evidence.

## Introduction

Crohn's disease is a chronic, systemic, and relapsing-remitting disease that can affect any part of the gastrointestinal (GI) tract, from the oral cavity to the anus. This case report highlights an atypical presentation of new-onset CD, featuring a benign abdominal mass. This unique presentation underscores the diverse manifestations of the disease. In children, the causes of abdominal masses are extensive, ranging from infectious to hereditary and benign to neoplastic, often originating from organs within the intra-abdominal cavity, including the GI, genitourinary, and endocrine systems. However, their etiology, presentation, and age-related characteristics can vary (1). Typical presentations of CD include diarrhea, abdominal pain, oral ulcers, growth deceleration, weight loss, anorexia, hematochezia, perianal disease, fistula, abscess formation, and extraintestinal manifestations. Management of such cases typically involves multiple subspecialties, including surgery, hematology/oncology, endocrinology and gastroenterology, to achieve better management outcomes.

## Case presentation

A previously healthy 12-year-old female experienced mild RLQ pain six months prior to the clinic visit. The pain resolved on its own within a few days. Shortly after this episode, she noticed a mass in the RLQ. During a well-child visit, her primary care physician performed a sonogram, which revealed an indeterminate 2 cm × 3 cm solid mass in the RLQ and enlarged mesenteric lymph nodes (MLN). The patient did not report any gastrointestinal symptoms, and her review of systems was unremarkable. On physical examination, a palpable 3 cm × 3 cm hard, mobile mass was noted in the RLQ. The remainder of the examination was normal. The patient's biological mother has a history of questionable ulcerative colitis and has not been on any treatment for 13 years.

## Diagnostic workup

**Figure 1:**
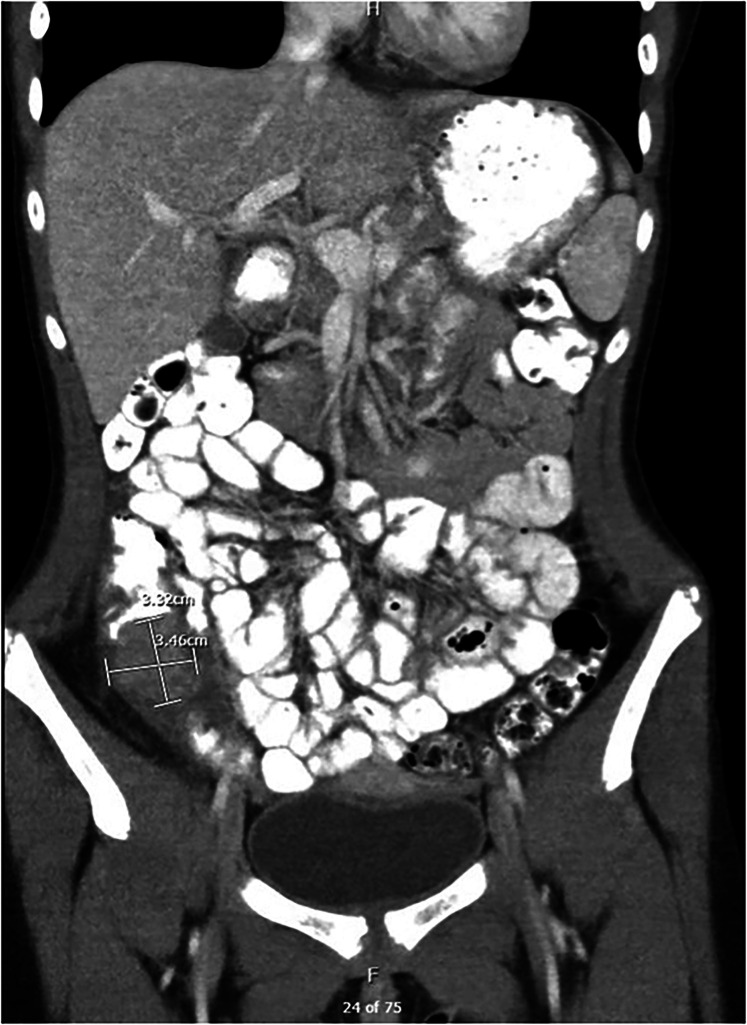
CT scan: intraluminal mass.

**Figure 2:**
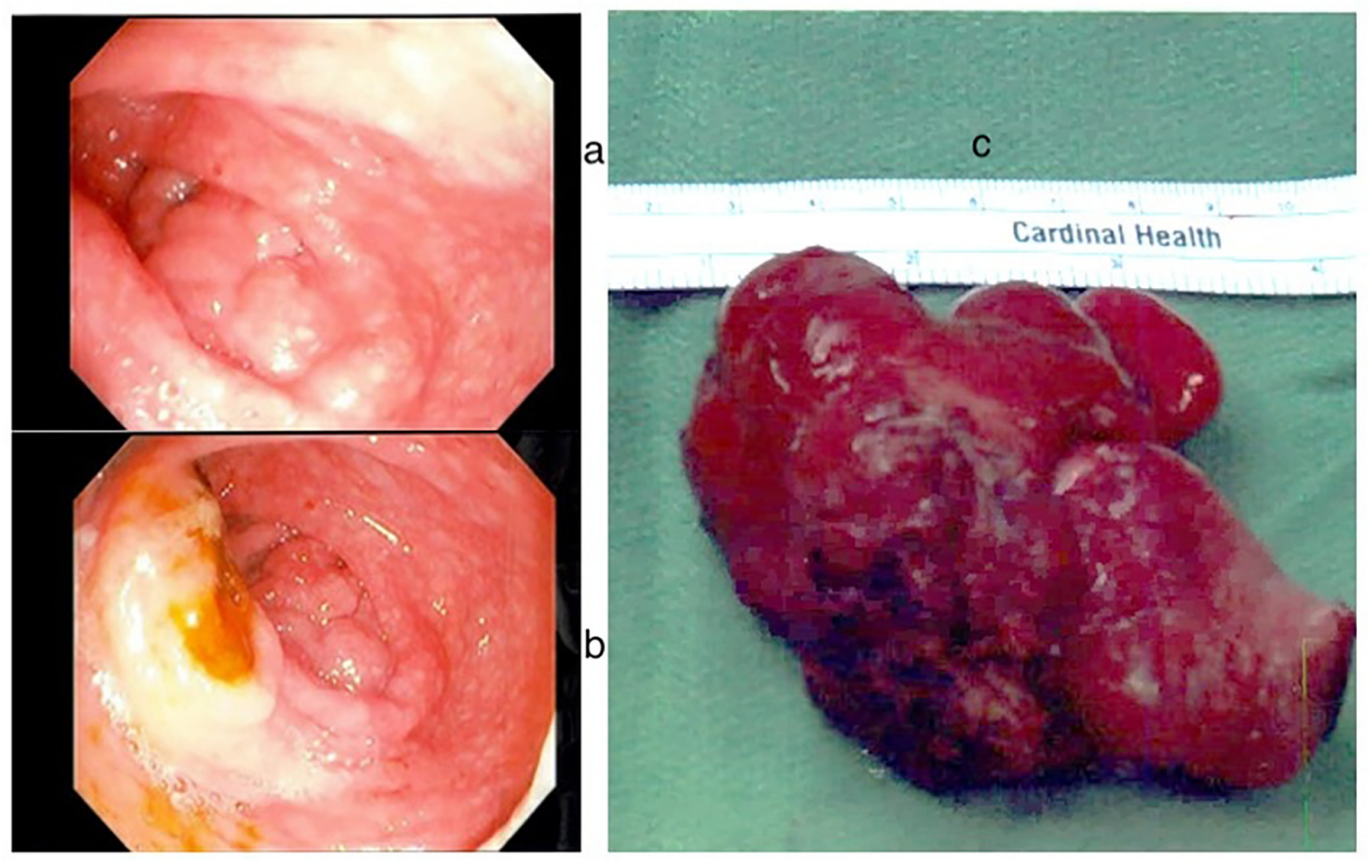
Endoscopic findings: **(a)** polypoid mass in the cecum originating from appendix **(b)** polypoid mass in the cecum and ileocecal valve **(c)** gross surgical specimen.

**Figure 3:**
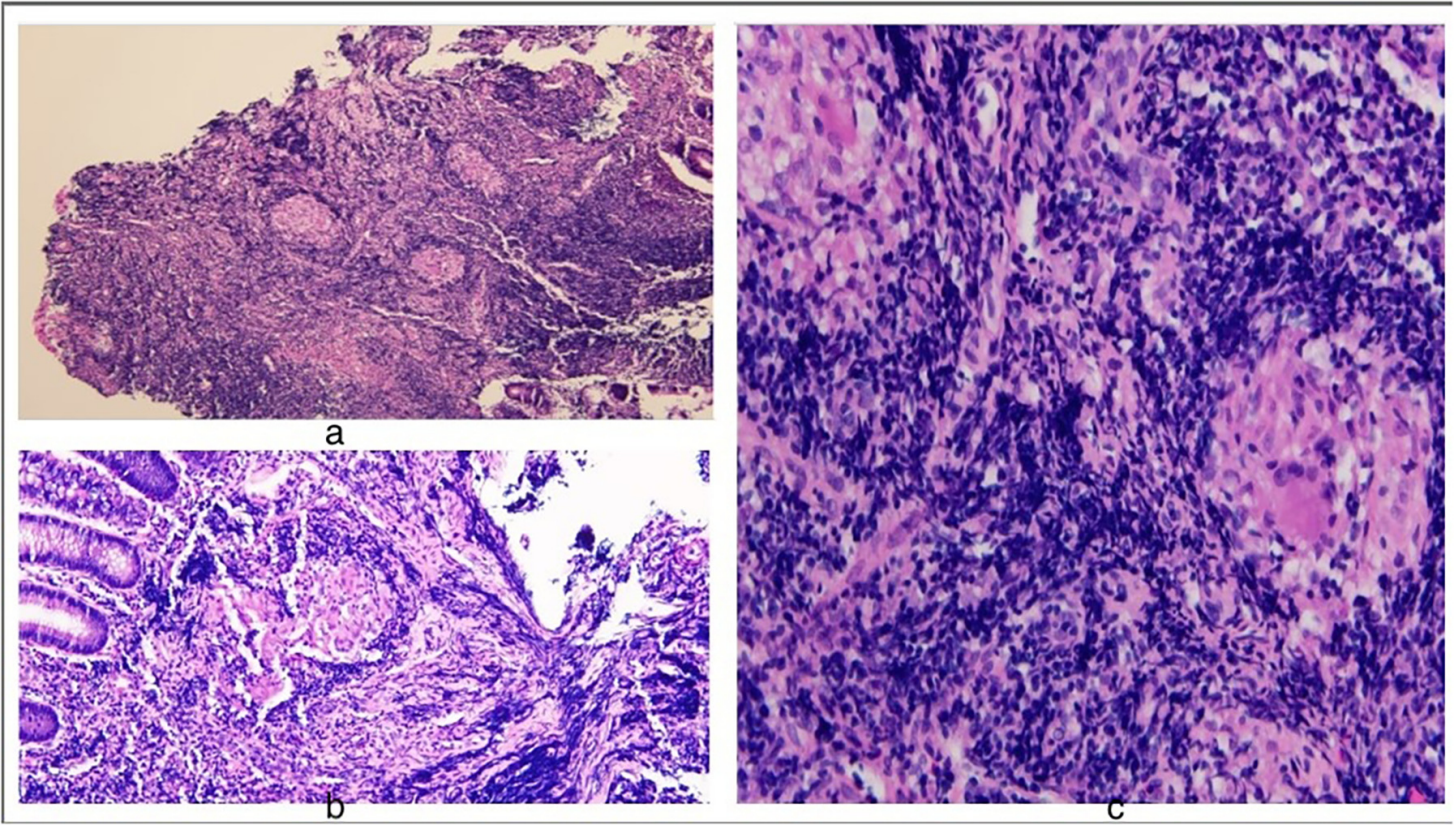
Histology findings: **(a)** H&E stain 10× acute and chronic colitis and non-caseating granuloma **(b)** 40× crypt architectural changes **(c)** 40× epithelioid non-caseating granuloma with multinucleated giant cell macrophage and inflammatory cells.

• Laboratory Tests (Labs): Results indicated mild anemia with a hemoglobin level of 11.7 g/dl, an elevated erythrocyte sedimentation rate (ESR) of 17 mm/h and calprotectin level of 407 μg/g (normal <50)
• Ultrasound (US) Abdomen: Revealed an indeterminate 2 cm × 3 cm solid mass-like lesion with a concentric appearance, along with multiple enlarged MLN.
• CT Enterography: Identified a 3.5 cm × 3.3 cm × 2.8 cm soft tissue-enhancing cecal mass appearing intraluminal (Figure 1).
• Esophagogastroscopy: Both endoscopic and histologic findings were unremarkable.
• Colonoscopy: Detected a polypoid mass in the cecum, seemingly originating from the appendix area, with erythematous and superficially eroded surfaces (Figure 2). The terminal ileum (TI) and the rest of the colon appeared normal. Biopsies showed intestinal mucosa with non-caseating granulomas and increased inflammation, including lymphocytes, plasma cells, and eosinophils. No architectural distortion. Acid-fast and fungal stains were negative. Malignancy could not be ruled out from submucosal biopsies.
• Surgery 01/2023: Ileocecectomy with primary anastomosis was performed. The subsequent biopsies exhibited findings of a benign appendix with phlegmonous inflammation, florid submucosal lymphoid follicular proliferation, focal intramucosal non-caseating granulomas, and associated mucosal hyperplasia. Scattered clusters of giant cells, suggestive of microperforation with healing. Pronounced serosal adhesions The lymph nodes displayed focal non-necrotizing granulomas but did not exhibit any evidence of malignancy (Figure 3).
• Prometheus IBD Panel 03/2023: Positive for perinuclear antineutrophil cytoplasmic antibodies (p-ANCA). Significant for mutations in genes such as ECM1, STAT3, ATG1BL, and NKX2.

### Follow up

Extensive discussion with the family about the natural course of penetrating CD, treatment options, and complications of untreated CD was held. The family opted not to pursue any therapeutic intervention at this time and agreed to repeat calprotectin levels serially. The patient is currently clinically doing well and asymptomatic with normal labs (CBC, CMP, ESR, CRP and calprotectin).

## Discussion

Intra-abdominal masses are frequently observed in children, presenting a wide range of potential diagnoses, both benign and malignant. These masses often mimic conditions with granulomatous, infectious, constipation-related, and neoplastic origins, all of which must be considered and excluded. Such conditions include adenocarcinoma, neuroendocrine tumors, sarcomas, gastrointestinal stromal tumors (GISTs), CD, histoplasmosis, tuberculosis, and sarcoidosis. Abdominal masses typically remain active but asymptomatic for prolonged periods. New-onset CD presenting as a large cecal mass is a rare presentation and complication. While such presentations are uncommon, a few reported pediatric cases have exhibited similar initial mass-like presentations, with the majority ultimately turning out to be malignancies and only a few cases diagnosed as CD.

Crohn's disease is a chronic systemic inflammatory disease of the gastrointestinal tract, with increasing prevalence and incidence, from fewer than 4 cases per 100,000 individuals in the 1970s to more than 7 per 100,000 in 2000s (2). About 20% of IBD cases present in childhood. IBD in children involves a complex interplay of environmental factors, microbial influences, and immune responses in genetically susceptible individuals. Children often display an aggressive phenotype, presenting complications such as fistulas, abscessed, strictures, and perianal disease. In CD, the transmural inflammation leads to deep ulcerations, which may result in perforation of the intestinal wall. The slow developing micro-perforation can form a contained abscess cavity, which could potentially evolve into a mass. Imaging, surgery, and pathology are crucial for identifying the cause and guiding further management. Early and aggressive treatment yields favorable outcomes once the diagnosis is confirmed and other conditions causing intra-abdominal masses are excluded.

Caricato et al. (2) reported a male patient with a history of CD who, after undergoing an ileocolic resection, presented with abdominal distension and a RLQ mass. En bloc resection and histology revealed a GIST. In another case, Grimberg et al. (3) reported a 15-year-old girl with new onset constipation, diarrhea, and hematochezia. A CT abdomen and diagnostic laparoscopy revealed a large, calcified pelvic mass adherent to the cecum, TI, and appendix. Similar to our case, an *en bloc* resection with an ileocecectomy with primary anastomosis was performed, with histology suggestive of chronic colitis with dystrophic calcification.

When evaluating patients with an abdominal mass and considering IBD, CD emerges as a more probable diagnosis than ulcerative colitis. A review of the literature reveals numerous cases in the adult population (typically aged 35–45) where incidental extraluminal intra-abdominal masses led to the diagnosis of IBD. However, such cases are relatively rare in children, particularly when the initial presenting symptom is an intra-abdominal mass. Moreover, diagnosing granulomas can further complicate matters, given their smaller, poorly organized, and denser nature without confluence or caseation in CD, as opposed to intestinal tuberculosis (4, 5).

## Conclusion

This case emphasizes the importance of considering IBD in the differential diagnosis of intraluminal masses, stressing the need for early recognition and treatment. It highlights the significance of a comprehensive evaluation incorporating symptoms, radiology, and histological evidence, underscoring the diverse manifestations of CD and the necessity for a multidisciplinary approach to management.

## Data Availability

The original contributions presented in the study are included in the article/Supplementary Material, further inquiries can be directed to the corresponding author.
